# Supplementary material for: 10-DEBC Hydrochloride as a Promising New Agent against Infection of Mycobacterium abscessus
Source: Int J Mol Sci. 2022 Jan 6;23(2):591. doi: 10.3390/ijms23020591 (PMC8775589; doi:10.3390/ijms23020591)
Supplement: Supplementary file 1 [file ijms-23-00591-s001.zip › supplementary data.pdf]

Supplementary Figure S1

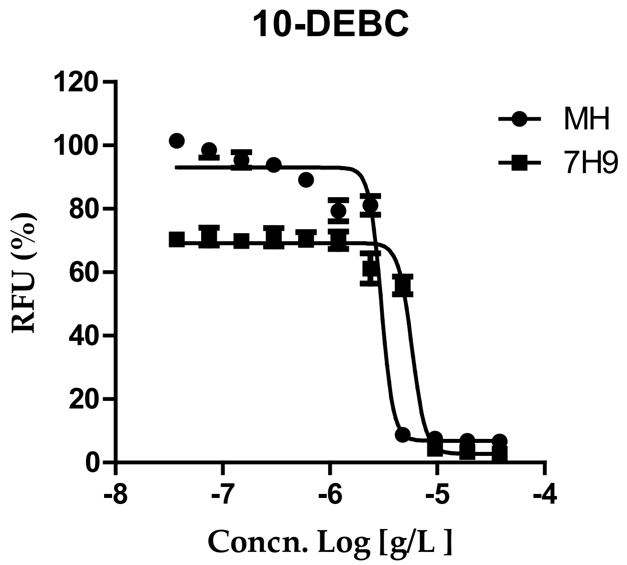

Supplementary Figure S2

A

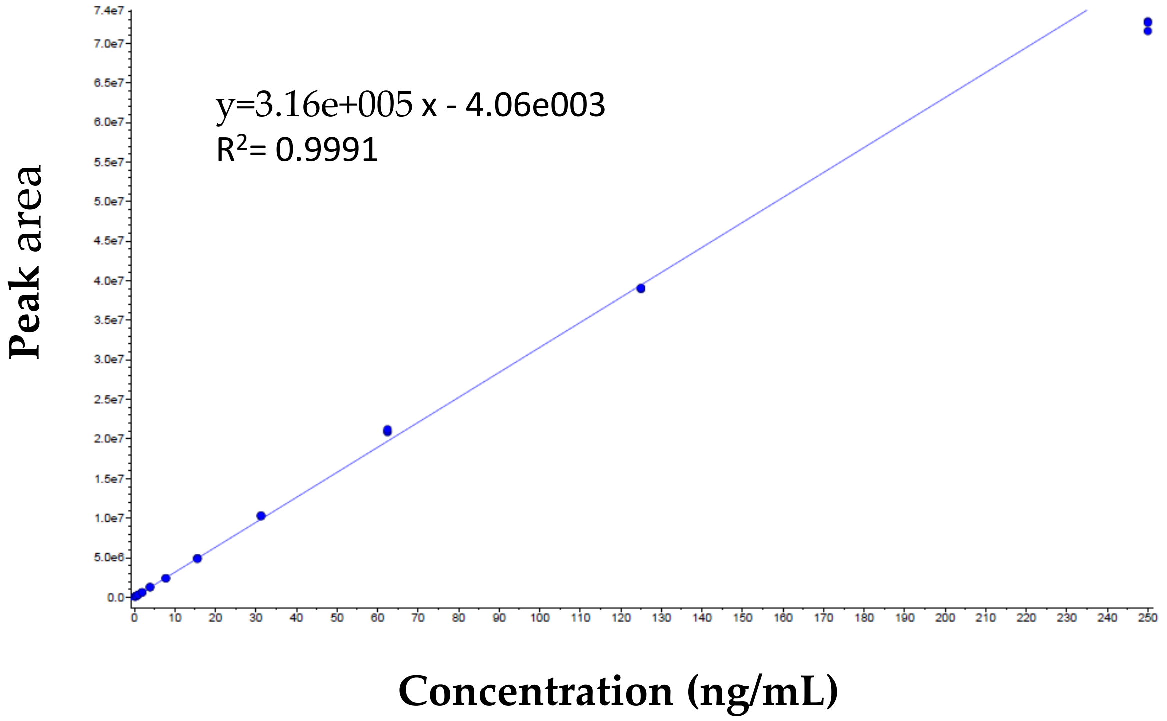

B

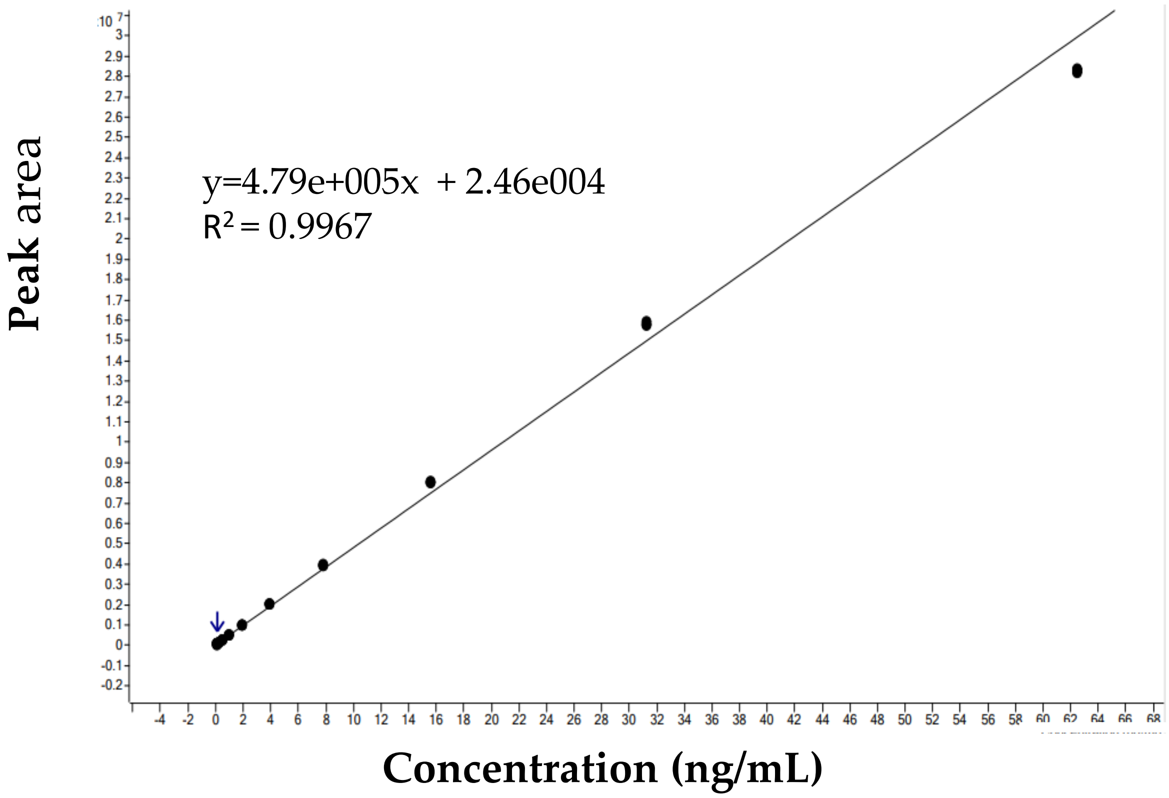

Supplementary Figure S2. Calibration curve. Signals for Clarithromycin were robust and linear between 0.244 and 250 ng/mL (A). Signals for 10-DEBC were robust and lin ear between 0.061 and 62.5 ng/mL (B).
