# Supplementary material for: 10-DEBC Hydrochloride as a Promising New Agent against Infection of Mycobacterium abscessus
Source: Int J Mol Sci. 2022 Jan 6;23(2):591. doi: 10.3390/ijms23020591 (PMC8775589; doi:10.3390/ijms23020591)
Supplement: Supplementary file 1 [file ijms-23-00591-s001.zip › Supplementary Table. MRM LIST_re.pdf]

Table S1. Mass spectrometer settings used for drugs

Drug	Precursor ion (m/z)	Product ion (m/z)	Fragmentor voltage(V)	Collision energy (V)
Clarithromycin	748.3	590.3	40	25
10-DEBC	345.1	237.1	166	20
